# An unusual occurrence: a case of venous thromboembolism in pregnancy associated with heterotaxy syndrome

**DOI:** 10.1186/s12878-015-0025-5

**Published:** 2015-05-12

**Authors:** Narendranath Epperla, Erika Peterson, Patrick Foy

**Affiliations:** Division of Hematology and Oncology, Medical College of Wisconsin, 9200 W Wisconsin Avenue, Milwaukee, WI USA; Division of Obstetrics and Gynecology, Medical College of Wisconsin, Milwaukee, WI USA

**Keywords:** Venous thromboembolism, Inferior vena cava, Low molecular weight heparin

## Abstract

**Background:**

Heterotaxy is a relatively uncommon congenital anomaly that is usually diagnosed incidentally on imaging studies in adults. We present an unusual case of venous thromboembolism in a 26 year old pregnant female with Heterotaxy syndrome.

**Case presentation:**

A 26 year-old pregnant female at 13 weeks gestation suffered cardiac arrest with successful cardiac resuscitation and return of spontaneous circulation. The cardiac arrest was secondary to massive pulmonary embolism requiring thrombolytic therapy and stabilization of hemodynamics. She had extensive evaluation to determine the etiology for the pulmonary embolism and was noted to have an anatomic variation consistent with heterotaxy syndrome on imaging studies. After thrombolysis the patient was treated with UFH and then switched to enoxaparin without complication until 25 weeks of gestation when she experienced worsening abdominal pain with associated headaches, lightheadedness and elevated blood pressures needing elective induction of labor. The infant died shortly after delivery. The anticoagulation was continued for additional 3 months and she was subsequently placed on low dose aspirin to prevent recurrent venous thromboembolic episodes. She is currently stable on low dose aspirin and is into her third year after the venous thromboembolism without any recurrence.

**Conclusion:**

To our knowledge, this is the first reported case of venous thromboembolism in pregnancy associated with heterotaxy syndrome. A discussion on pathophysiology of venous thromboembolism in pregnancy and heterotaxy syndrome has been undertaken along with treatment approach in such situations.

## Background

Heterotaxy is an uncommon polymalformative syndrome characterized by congenital anomalies that arise from disorderly arrangement of asymmetric thoracic and abdominal viscera and blood vessels [1]. The estimated prevalence is approximately 1 in 10,000 live births [2]. Morbidity and mortality rates are high approaching nearly 70 % due to major cardiac malformations (especially in heterotaxy with asplenia patients). Five to ten percent of patients with minor or no cardiac anomalies will survive to the adulthood [3–5]. Some will remain asymptomatic and can be diagnosed incidentally on imaging studies. Heterotaxy has been reported to be associated with VTE and is felt to be related to abnormal lower extremity venous system [6]. Infact in the last few years some authors considered it as a VTE risk factor. However VTE in pregnancy with heterotaxy syndrome has not been reported.

Herein we present a case of 26 year-old pregnant female who suffered cardiac arrest related to massive pulmonary embolism requiring cardiac resuscitation. Imaging studies incidentally discovered anatomic variation consistent with heterotaxy syndrome. She was anticoagulated for 6 months with low molecular weight heparin (LMWH) and subsequently placed on low dose aspirin to prevent recurrent VTE. A detailed discussion of the pathophysiology of VTE in pregnancy and heterotaxy syndrome has been undertaken with a focus on treatment approach in such situations.

## Case presentation

A 26 year old otherwise healthy, G1P0 female at 13 weeks gestation was admitted to medical intensive care unit after she experienced cardiac arrest at home. She had reported shortness of breath earlier that day and then collapsed. During initial paramedic evaluation, she was pulseless and found to be in pulse less electrical activity (PEA). She was successfully resuscitated with return of spontaneous circulation (ROSC) in approximately 7 min and was brought to the hospital. Emergency department (ED) evaluation revealed the patient to be nonresponsive, and she was immediately intubated. Her exam was remarkable for cold and cyanotic extremities, without palpable distal pulses but strong femoral pulse. Shortly after her arrival to ED, the patient became pulse less again needing resuscitation and ROSC on 2 occasions.

**Fig. 1:**
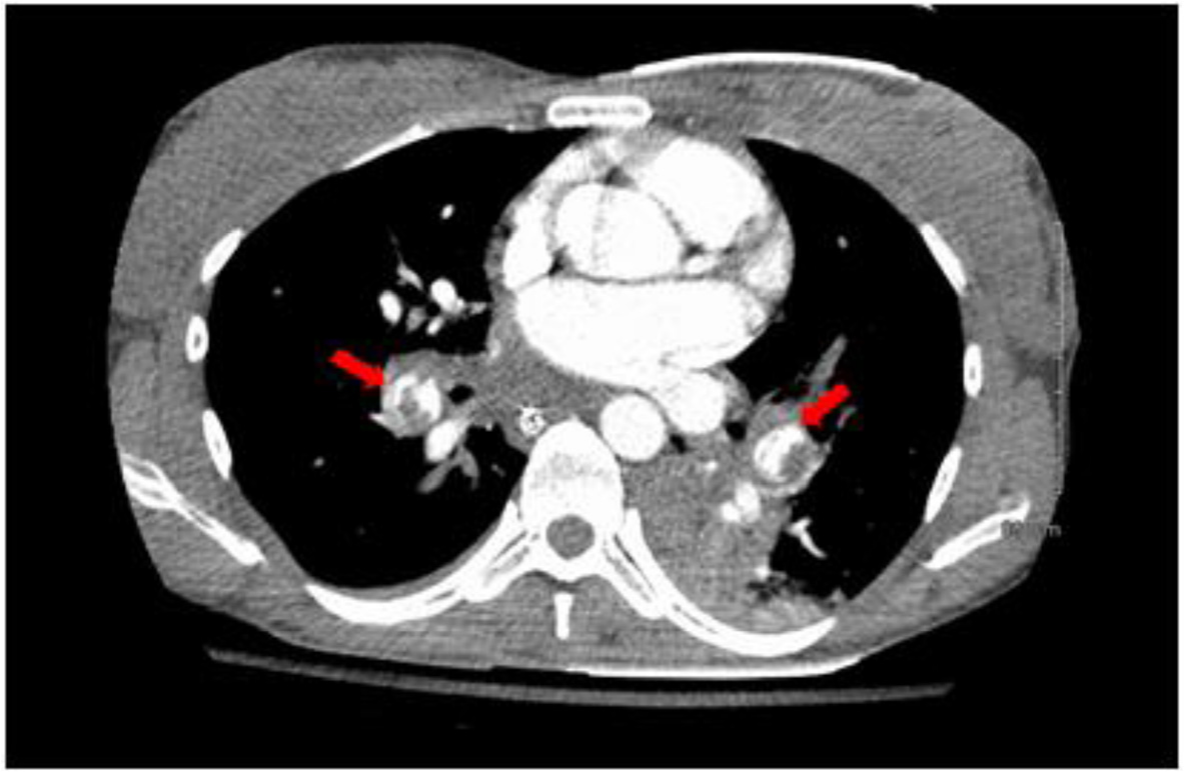
CT scan of the chest with contrast. The red arrows depict bilateral near occlusive pulmonary emboli

**Fig. 2:**
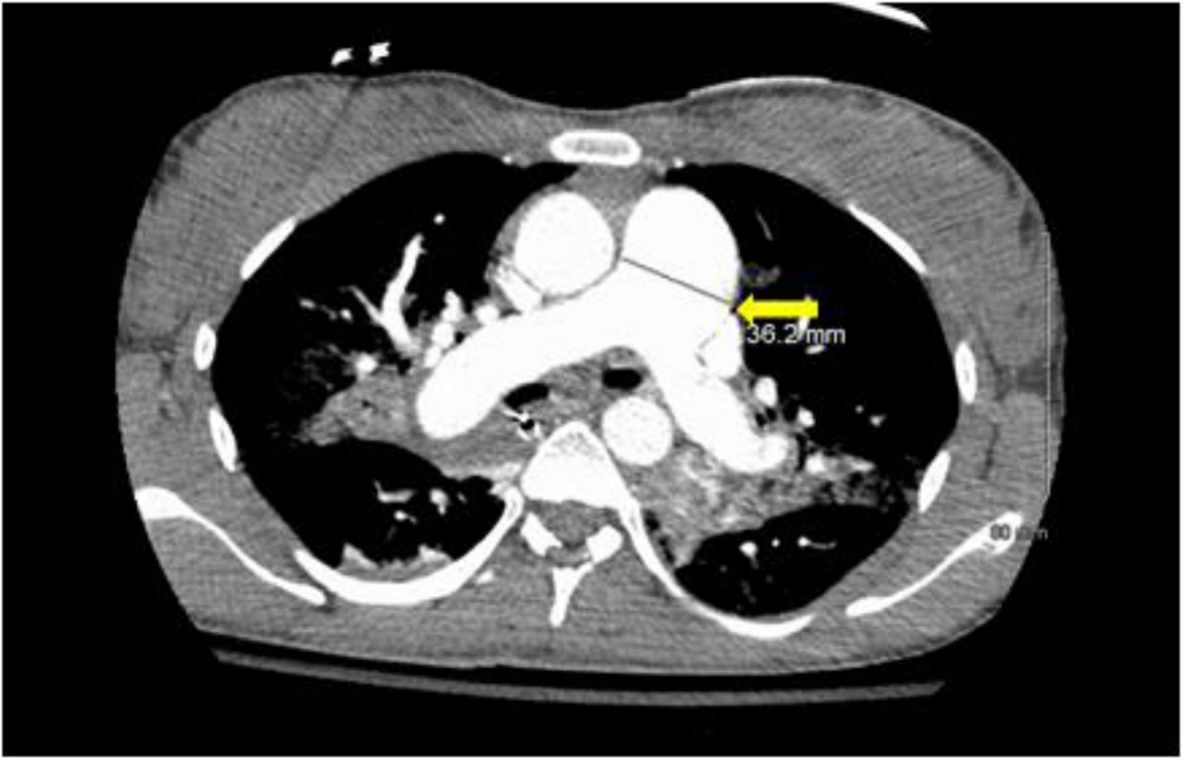
CT scan of the chest with contrast. The yellow arrow depicts enlarged main pulmonary arterial trunk

Initial blood work was remarkable for severe metabolic acidosis (HCO3 of 10) and elevated troponin I (2.77 ng/ml, normal range 0–0.34 ng/ml). EKG showed right bundle branch block while bedside 2 D echocardiogram demonstrated right heart strain pattern. Bedside ultrasound (USG) showed no significant free fluid with live intrauterine gestation. Bilateral lower extremity compression venous dopplers of the entire venous system were negative for any evidence of deep venous thrombosis. She underwent computerized tomography (CT) of the chest which revealed a nearly occlusive thrombus in the bilateral lower lobar arteries extending into the segmental arteries (Fig. 1). The main pulmonary arterial trunk was noted to be enlarged with evidence of right heart strain (Fig. 2). Head CT scan was negative for any acute intracranial abnormalities. She was administered intravenous tissue plasminogen activator (TPA) (50 mg after ROSC the second time) in the ED with improvement in her hemodynamics. Hypothermia protocol was concurrently initiated. She was subsequently transferred to medical intensive care unit on unfractionated heparin (UFH), bicarbonate and norepinephrine drips. The patient was extubated two days following and was gradually weaned off blood pressure support. UFH drip was titrated based on aPTT and 6 days later she was transitioned to LMWH (dalteparin 10,000 units subcutaneously once daily in view of her insurance issues). She had aggressive supportive care and after a 2 week hospital stay, she was discharged to rehabilitation facility on Dalteparin. A month later dalteparin was changed to enoxaparin 55 mg (1 mg/kg) s/q twice daily (monitored with anti Xa levels).

At approximately 20 week’s gestation, the patient had a fetal ultrasound that revealed singleton gestation with massive hydrops, fetal contractures and ventriculomegaly/hydranencephaly. It was presumed that these findings were secondary to early hypoxic injury and she was counseled about poor prognosis. During her 25th week gestation, the patient noted worsening abdominal pain with associated headaches, lightheadedness and weakness. She had elevated blood pressures and was suspected to have mirror syndrome. Enoxaparin was discontinued 24 h prior to the planned procedure with initiation of UFH drip. The patient underwent elective induction of labor with palliative care services. The infant died shortly after delivery. The patient experienced increased vaginal bleeding secondary to retained products of conception and underwent dilatation and curettage with achievement of hemostasis. Enoxaparin at previous dosage (55 mg s/q twice daily) was restarted 24 h after the delivery without any major or untoward complication. Platelet count and anti-Xa levels were checked while the patient was on enoxaparin. Anti-Xa levels were in therapeutic range (0.8–1, goal 0.6–1.0 IU/ml) and platelet counts remained within normal limits.

**Fig. 3:**
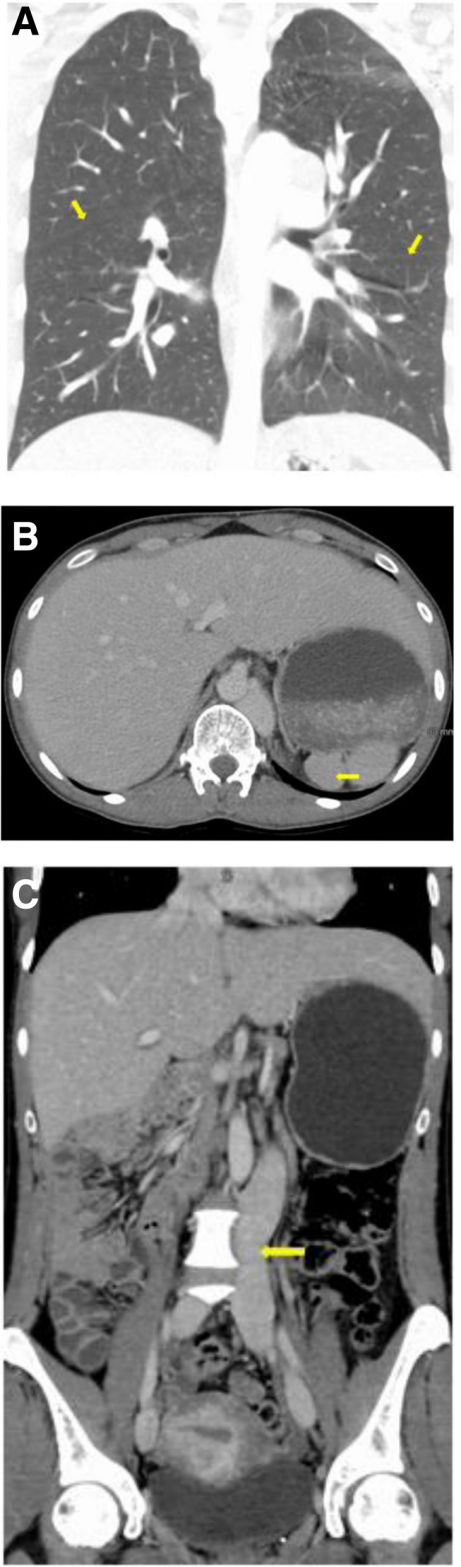
**A**. CT scan of the chest with contrast. Coronal section showing bilateral bilobed lungs (yellow arrows depict the major fissure). **B**. CT scan of the abdomen. The arrow points to multiple splenic lobules suggestive of polysplenia. **C**. CT scan of the abdomen. Coronal section showing azygous continuation of inferior vena cava

In order to identify the cause of her VTE the patient had further work up including thrombophilia testing and CT chest, abdomen/pelvis. Thrombophilia testing revealed the patient to be wild type for Factor V and Prothrombin gene. Protein C and S antigen levels were normal. Antithrombin activity was initially low (71, normal range 75–125 % ACT) at the time of initial VTE but subsequently normalized (107). Antiphospholipid antibody testing including lupus anticoagulant, anti-cardiolipin antibodies and beta 2 glycoprotein I antibodies was negative. CT chest, abdomen and pelvis showed constellation of imaging findings compatible with heterotaxy syndrome. These included left-sided superior vena cava (SVC) draining into the left coronary sinus, hemiazygous vein drains into left SVC, absent azygous vein, absent (interrupted) inferior vena cava (IVC) below the hepatic IVC with persistent left IVC which drains into left coronary sinus, polysplenia, bilateral left lung and benign hepatic hemangioma (Fig. 3A-C).

The patient completed 6 month duration of anticoagulation with LMWH and was subsequently placed on ASA 81 mg. Prior to discontinuation of anticoagulation therapy the patient had CT pulmonary angiogram which showed normal diameter of the main pulmonary artery without any evidence of acute or chronic pulmonary emboli. She is currently stable on low dose ASA and is into her third year after the VTE without any recurrence. She has had no further pregnancies.

## Discussion

Heterotaxy or situs ambiguous refers to malposition and dysmorphism of viscera and blood vessels, often with indeterminate atrial arrangement. This abnormal arrangement of body organs is different from the orderly arrangement seen in situs solitus or situs inversus. Heterotaxy syndrome can be associated with either asplenia or polysplenia. Our patient had heterotaxy with polysplenia and hence will limit our discussion to this abnormality and hereon will be referred to as heterotaxy.

Heterotaxy implies that the patients have bilateral bilobed lungs, bilateral pulmonary atria, a centrally located liver, a stomach in indeterminate position, interrupted IVC with azygous continuation and multiple spleens [1]. After polysplenia, the most frequent finding in heterotaxy is the hypoplasia of the IVC with absence of the intrahepatic segment and direct continuation with the azygous venous system. Heterotaxy commonly occurs as a sporadic condition but it can be inherited [7].

Varied timing of embryological causative factors during the development of fetus can explain the wide range of anomalies in the heterotaxy syndrome. Cardiac anomalies are mostly the result of abnormal embryological development around day 28 of embryogenesis, as it is during this period that connection between primitive heart and venous channels occurs [8]. The failure of fusion of fetal lobules leads to individualization of multiple splenic nodules, which are located along the greater curvature of the stomach, as the spleen develops in the mesogastric region [9, 10]. IVC is a complex vascular structure that is developed during weeks 6–8 of embryogenesis [11]. During this period three pairs of primitive venous channels (posterior cardinal, subcardinal and supracardinal veins) form the mature venous system (IVC) through a complex sequential process. Various malformations including partial or even complete absence of IVC can result from the failure in the developmental steps related to embryonic dysontogenesis that can affect separate segments or even the entire IVC [12–14]. Thus mutations in genes that control left-right patterning and teratogenic exposures in early embryonic period underlie majority of heterotaxy cases [15].

The occurrence of DVT seems to be higher in patients with heterotaxy compared to the general population. It is postulated that stasis related to the abnormal venous drainage of the lower extremity venous system due to interrupted IVC [16, 17] and increased platelet aggregation related to the anomalous drainage of the splenic venous system into the azygous system seems to be the possible culprits for heightened risk of pulmonary thromboembolism in these patients [9, 18].

VTE is 5 times more frequent in pregnant women than in non-pregnant women of similar age [19]. This is because pregnancy induces a state of venous stasis and hypercoagulability from a combination of physical, hormonal and hematological changes leading to an increased risk for VTE. Venous stasis occurs from progesterone mediated increased venous distension in the first trimester and the compressive effect by the enlarging uterus on the common iliac vein in the late second and third trimesters [20, 21]. In addition there is an imbalance between the procoagulant (increased circulating levels of fibrinogen, von Willebrand factor, VII, VIII, IX, X and XII and increased generation of fibrin) and anticoagulant factors (decreased Protein S levels and fibrinolysis) through the pregnancy [19–23]. Though pregnancy is considered to be a relative contraindication to systemic thrombolysis (recombinant TPA or streptokinase) in hemodynamically unstable patients with pulmonary embolism, the risk of complications for pregnant females treated with thrombolytic agents may be similar to that in the non-pregnant population [24].

In our case, the exact etiology for VTE is unclear. It may be related to pregnancy alone or mechanical factors from distorted venous system anatomy or a combination of both. Because the patient’s VTE occurred early in pregnancy, uterine enlargement is unlikely to have contributed to venous thrombosis. Effects of pregnancy in heterotaxy have been poorly described.

Challenges to the clinical management of our patient include the duration of anticoagulation and future pregnancy. Based on the 2012 American College of Chest Physicians (ACCP) guidelines, anticoagulation with LMWH is recommended for at least 3 months from the initial pulmonary embolism and 6 weeks postpartum [25]. In our case, the patient was into her 3rd month of anticoagulation when she had induction of labor and delivery; hence the anticoagulation was continued for additional 3 months for a total of 6 months. Subsequently she was placed on low dose aspirin (81 mg) to prevent recurrent VTE (especially given her heterotaxy syndrome) based on the Warfarin and Aspirin (WARFASA) and Aspirin to Prevent Recurrent Venous Thromboembolism (ASPIRE) trials as well as the individual patient data analysis of WARFASA and ASPIRE trials performed by the INSPIRE Collaboration [26–28]. Currently there is no evidence about the risk of VTE recurrence and anticoagulation duration in similar patients. According to the 2012 ACCP guidelines the decision regarding the duration of anticoagulation in a patient should be made after careful evaluation of both the risk of VTE recurrence and bleeding risk. Given the congenital prothrombotic risk factor (interrupted IVC related to heterotaxy syndrome), severity of the presentation and apparently low bleeding risk extended anticoagulation treatment with targeted oral anticoagulation maybe a suitable alternative to aspirin with periodic assessment of the bleeding risk.

Our patient had a massive pulmonary embolism with cardiac arrest that created a great sense of apprehension both in the patient and her family regarding future pregnancy. Unfortunately, there is paucity of data to either support or refute future pregnancy associated with her condition. Previous VTE alone is not a contraindication to future pregnancy provided anticoagulation is available. However patients with heterotaxy syndrome and VTE are undoubtedly at increased risk of VTE compared to other pregnant women. Hence future pregnancies in this patient group should be considered high-risk, and requires multi-disciplinary management. We recommend full intensity anticoagulation with LMWH (likely enoxaparin 1 mg/kg two times daily) with regular monitoring of anti-Xa levels prior to her pregnancy. It is important to measure anti-Xa levels 4 h after the last dose and be aware of the different targets based on which LMWH regimen is used (once-daily [goal 1.0–2.0 IU/mL] or twice-daily [goal 0.6–1 IU/mL]). In addition we recommend that there is a detailed discussion between the physician and the patient regarding the role of thrombolytic therapy in the event of recurrent VTE and possible termination of pregnancy in the worst case scenario.

## Conclusion

To our knowledge this is the first reported case in the English literature that shows pregnancy associated VTE in a person with heterotaxy. After completion of anticoagulation therapy for the initial thrombotic event, the patient needs secondary prevention for VTE recurrence and low dose aspirin was the chosen treatment in this case. Although there are no published guidelines we believe that there are no contraindications for future pregnancy in this special group provided the patient is on anticoagulation prior to the pregnancy.

## Consent

Written informed consent was obtained from the patient for publication of this case report and any accompanying images. A copy of the written consent is available for review by the Editor-in-Chief of this journal.

## Abbreviations

VTE: Venous thromboembolism
LMWH: Low molecular weight heparin
IVC: Inferior vena cava
CT: Computerized tomography
ASA: Aspirin
